# Molecular Properties of Guar Gum and Pectin Modify Cecal Bile Acids, Microbiota, and Plasma Lipopolysaccharide-Binding Protein in Rats

**DOI:** 10.1371/journal.pone.0157427

**Published:** 2016-06-17

**Authors:** Tannaz Ghaffarzadegan, Nittaya Marungruang, Frida Fåk, Margareta Nyman

**Affiliations:** Food for Health Science Centre, Lund University, Lund, Sweden; University Medical Center Utrecht, NETHERLANDS

## Abstract

Bile acids (BAs) act as signaling molecules in various physiological processes, and are related to colonic microbiota composition as well as to different types of dietary fat and fiber. This study investigated whether guar gum and pectin—two fibers with distinct functional characteristics—affect BA profiles, microbiota composition, and gut metabolites in rats. Low- (LM) or high-methoxylated (HM) pectin, and low-, medium-, or high-molecular-weight (MW) guar gum were administered to rats that were fed either low- or high-fat diets. Cecal BAs, short-chain fatty acids (SCFA) and microbiota composition, and plasma lipopolysaccharide-binding protein (LBP) levels were analyzed, by using novel methodologies based on gas chromatography (BAs and SCFAs) and 16S rRNA gene sequencing on the Illumina MiSeq platform. Strong correlations were observed between cecal BA and SCFA levels, microbiota composition, and portal plasma LBP levels in rats on a high-fat diet. Notably, guar gum consumption with medium-MW increased the cecal amounts of cholic-, chenodeoxycholic-, and ursodeoxycholic acids as well as α-, β-, and ω-muricholic acids to a greater extent than other types of guar gum or the fiber-free control diet. In contrast, the amounts of cecal deoxycholic- and hyodeoxycholic acid were reduced with all types of guar gum independent of chain length. Differences in BA composition between pectin groups were less obvious, but cecal levels of α- and ω-muricholic acids were higher in rats fed LM as compared to HM pectin or the control diet. The inflammatory marker LBP was downregulated in rats fed medium-MW guar gum and HM pectin; these two fibers decreased the cecal abundance of *Oscillospira* and an unclassified genus in *Ruminococcaceae*, and increased that of an unclassified family in *RF32*. These results indicate that the molecular properties of guar gum and pectin are important for their ability to modulate cecal BA formation, gut microbiota composition, and high-fat diet induced inflammation.

## Introduction

Bile acids (BAs) act as signaling molecules in a number of physiological processes associated with host health [1, 2]. For instance, some BAs have been linked to diet-induced obesity and the development of insulin resistance, while others are associated with carcinogenesis in the colon [1, 3]. Thus, BA profiles serve as a measure of physiological homeostasis. Gut microbiota regulate the composition of BAs via deconjugation and dehydroxylation reactions, and can thereby modulate their signaling functions [2]. Some bacterial strains are more active than others in the production of specific secondary BAs [4]. However, the relationship between BAs and the gut microbiota is complex, with BAs altering microbiota composition by acting as a substrate [5]. One study found that gut microbiota composition was altered in rats fed a single BA (cholic acid, CA) in a manner that was similar to those consuming a high-fat diet [6]. Supplementation with very low amounts of chenodeoxycholic acid (CDCA) also changed BA metabolism, highlighting the importance of diet in this process [7]. Other factors also influence the composition and metabolic activity of colonic microbiota: availability of nutrients—particularly dietary fiber (DF) and to some extent protein—is important for the formation of short-chain fatty acids (SCFAs), with different types of DF giving rise to different SCFA amounts and profiles during colonic fermentation [8]. It is therefore of interest to determine whether BA composition can be modulated by diet, particularly in relation to SCFAs derived from fermentable ingested DF.

When a meal is consumed, BAs are secreted from hepatocytes and are absorbed in the duodenum by passive diffusion, or are actively transported from the terminal ileum to the liver via the portal vein to complete enterohepatic recirculation [1]. CA and CDCA are the primary BAs formed from cholesterol by the liver in humans, whereas α-, β-, and ω-muricholic acids (MCA) are present only in rodents and are responsible for cholesterol clearance from the body [3]. Once in the colon, BAs are metabolized by microbiota and transformed into secondary BAs including deoxycholic and lithocholic acid (DCA and LCA, respectively), which are derived from CA and CDCA, respectively. Ursodeoxycholic acid (UDCA) is also present in the colonic environment [3] and inhibits DCA-induced apoptosis in cancer cells by disrupting classical apoptosis pathways and downregulating mitochondrial genes that facilitate cancer cell growth [9–11]. Moreover, UDCA is used as a drug to dissolve cholesterol gallstones and for treatment of liver dysfunction and primary biliary cirrhosis, and has been proposed for the treatment of type 2 diabetes [3, 12, 13]. Interestingly, mice fed a high-fat diet showed an increase in intestinal permeability that was inversely proportional to the level of fecal UDCA [14]. Notably, DCA induces intestinal inflammation and increases bile lithogenicity, i.e., the supersaturation and precipitation of cholesterol in bile [15]; this effect is counteracted by CDCA, which has also been used to dissolve gallstones [3, 16]. Less is known about hyodeoxycholic acid (HDCA), although it has been shown to decrease cholesterol absorption and improve high density lipoprotein function in low density lipoprotein receptor knockout mice [17] while its conjugated form is reportedly cytotoxic [18].

DFs are implicated in various aspects of metabolic syndromes, such as body weight gain, dyslipidemia, hypertension, insulin sensitivity, and levels of pro- and anti-inflammatory factors [19]. Water-soluble and viscous DFs such as pectin and guar gum decrease cholesterol levels in humans and animal models, often in a manner directly proportional to their viscosity in the small intestine. The molecular weight (MW) and degree of methoxylation of pectin are important for trapping of BAs by the DF network in the gut. Cross-linking by calcium ions and pectin molecules may also contribute to the formation of these networks, with high-methoxylated pectins binding lower amounts of BAs than the low-methoxylated forms [20]. Another mechanism that could affect BA profiles is SCFA formation. Guar gum and pectin degradation by colonic bacteria generates bacterial metabolites, mainly SCFAs. Colonic SCFA profiles are influenced by DF composition, glycosidic linkage types, and chain length [8]. Other factors influencing the SCFAs formed in colon are e.g. transit time, substrate viscosity and type of bacteria present. Guar gum yields high amounts of propionic acid whereas pectin yields acetic acid in the cecum of rats [21], which influence the BA profile [22].

Lipopolysaccharide (LPS)-binding protein (LBP) is an acute-phase glycoprotein synthesized by hepatocytes in response to a systemic rise in the level of LPS, a compound that is present in the outer membrane of Gram-negative bacteria. Binding of cluster of differentiation 14 receptor to LPS–LBP complexes activates the immune response [23]. Thus, a shift in gut microbiota composition towards more Gram-negative bacterial species may induce systemic inflammation. Gut microbiota composition may also affect the types of SCFAs that are formed. Butyric acid is the most physiologically significant of these since it increases the nutritional status of the mucosa, resulting in decreased permeability.

The aim of the present work was to determine whether guar gum and pectin with distinct functional properties [guar gum with three different MWs and pectin with two degrees of methoxylation (LM and HM)] can alter BA profiles, LBP level, and intestinal microbiota composition in the cecum of rats. We also investigated the correlation between analytical components (BAs and LBP), bacterial genera, and SCFA yield reported by a previous study [24]. Some BAs were found to be correlated with lipid levels in blood and liver in that study. It is known that the functional properties of DF determine the types of SCFA that are formed [25, 26]; however, ours is the first study to investigate the importance of these properties in relation to BA composition. It was previously shown that both guar gum and pectin affect cholesterol and blood glucose levels and liver steatosis, depending on viscosity and degree of methoxylation [24]. We report here that among the investigated DFs medium-MW guar gum and HM pectin had the greatest effects on cecal BA formation, gut microbiota composition, and high-fat diet-induced inflammation. This study shows the importance of molecular features of DF for the outcome of results and may explain the inconsistency sometimes reported in literature. Molecular properties of the DF should be considered when designing food with specific health effects.

## Materials and Methods

### Materials

Rats were fed pectin diets (Danisco, Norrköping, Sweden) with 24% or 70% methoxylation (LM and HM, respectively), or guar gum diets (Danisco) with low (315 kDa), medium (900 kDa), or high (2,000–3,000 kDa) MW that differed in terms of viscosity [24] (Table 1). Details of the composition of each diet can be found in Table A in S1 File.

**Table 1:** Experimental groups.*

**Diet**	**Fiber**
**Control**	None
**Pectin**	Low (24%) or high (70%) methoxylation
**Guar gum**	Low-, medium-, or high-MW

* Fat contents were the same for all diets—i.e., low fat (5%) or high fat (30%).

### Experimental design, diets, and preparation of cecal samples

Male Wistar rats with an initial weight of 129 ± 10 g were randomly divided into groups of seven animals each, and allowed to adapt to the environment for 6 days before the start of the 3-week experiment [24]. The groups were fed with different DF preparations (8% DF on a dry weight basis, DWB) along with a low- or high-fat diet (5% or 30% DWB, respectively) (Table 1). Rats fed with fiber-free wheat starch and 5% or 30% fat (DWB) served as controls for the low- and high-fat diets, respectively.

Each rat was given 12 g/day (DWB) of feed during the first 2 weeks of the experiment and 20 g/day in the final week. There were 12 groups in total: two were fed pectin (LM and HM), three were fed guar gum (three viscosities), and one was a fiber-free control group [24]. All diets were administered in the context of low and high fat. On day 21, animals were anaesthetized by subcutaneous injection (1:1:2, 0.15 ml/100g body weight) of a mixture of Hypnorm (Janssen Pharmaceutica, Beerse, Belgium), Dormicum (F. Hoffman-La Roche AG, Basel, Switzerland), and water. Cecal samples were taken from 84 rats (n = 7/group) and freeze-dried separately and stored at room temperature until BA analysis. Additionally, a section of cecum along with the contents was aseptically removed from each animal and stored at −80°C for bacterial genomic DNA analysis. Four or five samples from groups exhibiting the most pronounced effects were randomly selected for sequencing. A total of 14 animals fed a high-fat diet were selected for these analyses from the medium MW guar gum (n = 5), HM pectin (n = 4), and fiber-free control (n = 5) groups. The rats were healthy and active throughout the experiment, and the diets were well-tolerated. The Ethics Committee for Animal Studies (Review panel III) at Lund University approved the experiment (application number: M 56–12).

### Chemicals and reagents

BAs (CA, CDCA, DCA, LCA, UDCA, and HDCA) and 5β-cholanic acid (internal standard, IS) were purchased from Sigma-Aldrich (Steinheim, Germany), while α-, β-, and ω-MCA were obtained from Steraloids (Newport, RI, USA). Methanol (high-performance liquid chromatography grade), hydrochloric acid, and sodium hydroxide were from Merck (Darmstadt, Germany). Ultra-pure reagent water purified by a Milli-Q gradient system (Millipore, Bedford, MA, USA) was used in the experiments. Di-*n*-hexylether (DHE), *N*-methyl-*N*-(trimethylsilyl)trifluoroacetamide (MSTFA; derivatization grade), tri-*n*-octylphosphine oxide (TOPO), ammonium iodide (NH_4_I), and dithioerythritol (DTE) were supplied by Sigma-Aldrich.

### Analysis

#### BA extraction

Nine cecum BAs were analyzed by performing the recently-developed hollow-fiber liquid-phase microextraction method, followed by gas chromatography (GC), which has shown satisfactory linearity for all BAs [22]. To offset the matrix effect from cecum samples, a standard BA mixture (100 μg/ml) was used in the extraction process. Sodium hydroxide (0.01 M, 5 ml) was added to 20 mg cecum content and hydrolyzed for 1 h at 80°C, followed by addition of the standard. Five samples from each rat were protonated with HCl (0.01 M) to pH 6. Reagent water was added to obtain a final sample volume of 20 ml.

A double-ended, heat-sealed polypropylene hollow fiber membrane (200-μm wall thickness, 600-μm inner diameter, 0.2-μm pore size; Membrana, Wuppertal, Germany) was immersed in DHE containing 10% (w/v) TOPO for 1 h followed by the aqueous sample suspension for 2 h to obtain BAs by liquid membrane extraction. BAs were flushed out of the fiber lumen and IS (2 μl) and derivatization mixture (8 μl, MSTFA:NH_4_I:DTE) were added to 10 μl of acceptor solution obtained from each fiber membrane. The extract was injected onto a fused-silica capillary column (HP-ULTRA 1; J&W Scientific/Agilent, Santa Clara, CA, USA) coated with cross-linked methyl silicone (Ultra-1.25 M × 0.2-mm inner diameter, 0.33-μm film thickness). Cecal BAs were then analyzed by GC [22].

#### Analysis of gut microbiota composition by 16S rRNA gene sequencing

Cecal samples from high-fat groups of medium-MW guar gum (n = 5), HM pectin (n = 4), and fiber-free controls (n = 5) were analyzed for microbiota composition, since these groups showed the greatest changes in BA composition and inflammation. DNA was extracted from cecal tissue and contents by bead-beating, using the QIAamp DNA Stool Mini kit (Qiagen, Limburg, Germany). Forward (27F AGAGTTTGATCCTGGCTCAG) and reverse (534R ATTACCGCGGCTGCTGG) primers containing adapter sequences (Illumina, San Diego CA, USA) were used to amplify 16S rRNA genes (amplicon length, 507 bp) by PCR, with unique dual indices used to tag each PCR product [27]. Paired-end sequencing with a read length of 2 × 300 bp was performed on a Miseq instrument (Illumina), using a Miseq Reagent kit v3 (Illumina) at GATC Biotech (Konstanz, Germany).

#### Sequence analysis

Bacterial genomic 16S rRNA gene sequences were analyzed with the Quantitative Insights into Microbial Ecology (QIIME) software package, which enables analysis of high-throughput community sequencing data. Default parameters were used at each step except where specified [28]. Sequences were removed in the following cases: length < 200 nucleotides; those containing ambiguous bases, primer mismatches, or homopolymer runs > six bases; or those lacking the primer sequence. Forward and reverse reads were joined using Fastqjoin. After quality filtering, a total of 1,648,363 sequences and 712 operational taxonomic units (OTUs) were generated using the closed-reference OTU-picking method that ranged from 66,905 to 233,596 sequences/sample with a mean sequence count of 11,7740 per sample. Similar sequences were binned into OTUs using UCLUST [29], with a minimum pairwise identity of 97%. The most abundant sequence in each OTU was used to represent its bin. Representative sequences from each OTU were aligned using PyNAST (a python-based implementation of NAST in QIIME [30]) and taxonomy was assigned using the Greengenes [31] database (v. 13_5) and RDP classifier [32].

#### Quantification of plasma LBP

Plasma levels of LBP were determined by an LBP enzyme-linked immunosorbent assay kit for a wide variety of species (Hycult Biotech, Uden, Netherlands), according to the manufacturer’s instructions. Rat plasma samples were diluted 1:10 with dilution buffer prior to use in the analyses. The absorbance was read within 30 min at 450 nm in a SPECTROstar Nano Absorbance microplate reader (BMG Labtech, Ortenberg, Germany). Standard curves were generated using MARS data analysis software (BMG Labtech).

#### SCFAs and lipids in blood and liver

SCFAs in cecum [33] and cholesterol and triglyceride levels in liver and blood [34] were measured as previously described.

#### Statistical evaluation

The study design was randomized. Test diets containing three types of guar gum (low-, medium-, and high-MW), two types of pectin (LM and HM), and one fiber-free control administered in low- and high-fat settings were evaluated, yielding 12 test diets. Nine BAs were analyzed that were divided into three categories according to their classification and/or behavior when reporting/interpreting the results: a) primary BAs (CA, CDCA, and α-MCA); b) group I secondary BAs with possible preventative effects (UDCA, β-MCA, and ω-MCA); and c) group II secondary BAs that had opposite effects (LCA, DCA, and HDCA). The different groups were evaluated statistically both within and between low- and high-fat diet groups. In addition, when evaluating the BA data, diet treatments were grouped and the two fiber groups (LM+HM pectin and guar gum with low+medium+high MW) were compared to the fiber-free control group. Minitab statistical software (release 16.0; Minitab, State College, PA, USA) was used to analyze the effects of fat and DF. All BA results were analyzed with a general linear model (ANOVA) followed by Tukey’s test to determine the significant effects for each DF. Significant differences between low- and high-fat values were evaluated by Dunnett’s test. Results were considered significantly different at P < 0.05. LBP and metabolic marker data were evaluated by ANOVA followed by Tukey’s post-hoc test.

Prism 6 software was used to identify significant differences in the relative abundance of bacteria between groups by one-way ANOVA and Tukey’s correction for multiple comparisons at each taxonomic level. In addition, α and β diversities were analyzed in QIIME after rarefying the OTU table at 66,905 randomly selected sequences per sample for the entire data set, which included all samples used in the analysis.

Correlations between BAs, LBPs, SCFAs, and gut bacterial genera in groups fed medium-MW guar gum, HM pectin, or a fiber-free control diet in a high-fat setting were analyzed with SIMCA-14 software (Umetrics, Umeå, Sweden). A partial least squares (PLS) plot was used to illustrate correlations between the gut microbiota and different biomarkers. Pearson’s correlation was calculated for each pairwise combination of biomarkers and gut microbiota using Minitab17 and P values were corrected for multiple comparisons by the Benjamini-Hochberg procedure [35, 36]. Correlations between BAs and blood and liver cholesterol/triglyceride levels were evaluated with Pearson’s correlation.

## Results

### Metabolic data

**Fig 1:**
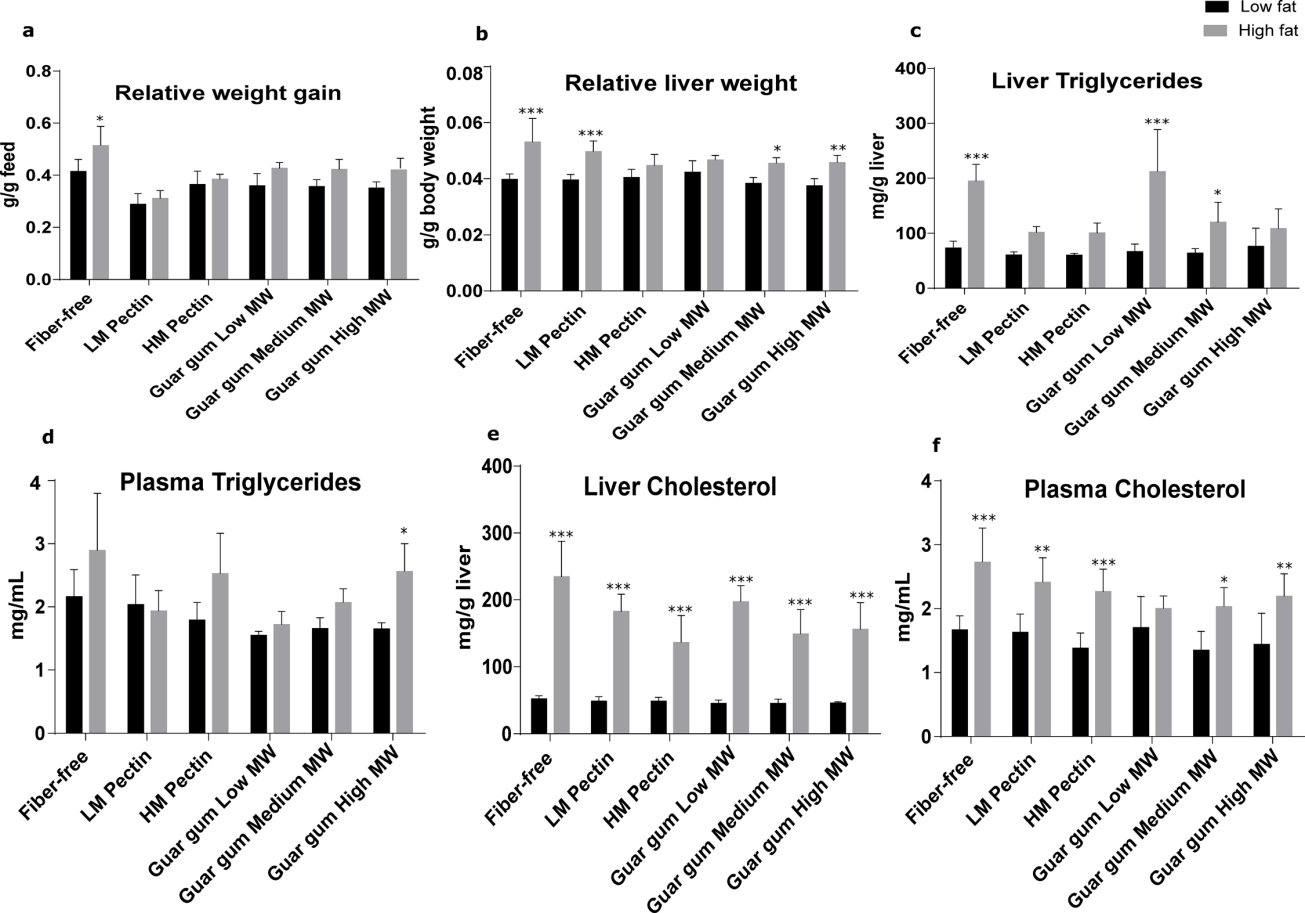
Levels of metabolic markers in rats. Data represent mean ± standard deviation of (a) relative body weight gain (g/g feed); (b) relative liver weight (g/g body weight); (c) liver triglycerides (mg/g liver); (d) plasma triglycerides (mg/ml); (e) liver cholesterol (mg/g liver); and (f) plasma cholesterol (mg/ml). *P < 0.05, **P < 0.01, ***P < 0.001 between low- and high-fat groups of rats fed the same fiber (one-way ANOVA with Tukey’s test for multiple comparisons).

Relative weight gain (g/g feed) was similar across groups, except for the group fed a diet containing no dietary fiber and high fat, which showed greater weight gain (P < 0.05) (Fig 1). The relative liver weight (g/g body weight) was higher in rats fed a high- as compared to a low-fat diet (P < 0.05–0.001 for all groups), except those fed HM-pectin and low-MW guar gum. Liver and plasma cholesterol levels were higher in rats fed a high-fat diet (P < 0.05–0.001). Liver and plasma triglyceride levels showed similar trends, but with fewer statistically significant differences.

### Effect of DF on total amounts of BAs in the cecum of rats

#### Low-fat diets

When molecular properties of guar gum and pectin were discounted (i.e., different levels of guar gum or pectin were grouped together), significant differences in primary BAs and group I secondary BAs were generally detected between groups, which showed higher values (except UDCA) with guar gum than with pectin or the fiber-free control diet (Table 2). The difference in group II secondary BAs between groups were less pronounced. However, the contribution of group II secondary BAs (LCA, DCA, and HDCA) was lower with DF in the diet (7–9%) than without (25%), while the opposite was true for group I secondary BAs (UDCA, β-MCA, and ω-MCA, 67–69% with DF vs. 52% without DF).

**Table 2:** Bile acids in the cecum of rats fed a fiber-free control, pectin, or guar gum diets in a low- or high-fat setting (μg/cecum).§

Fat	Dietary Fiber	n^1^	Primary BAs			Group I secondary BAs			Group II secondary BAs			Distribution of different types of BAs (%)^¥^
			CA	CDCA	α-MCA	UDCA	β-MCA	ω-MCA	LCA	DCA	HDCA	
**Low**	Fiber-free	6	174±31^a^	58±17^a^	190±20^a^	119±11^a^	465±133^a^	380±68^a^	54±11^a^	298±47^a^	101±14^a^	23:52:25
	Pectin	12	267±53^a^	102±22^a^^b^	262±48^a^	149±8^a^	640±94^a^	992±253^a^	35±6^a^	146±40^b^	70±14^a^	24:67:9
	Guar gum	21	576±20^b^	128±9^b^	466±39^b^	144±7^a^	1358±65^b^	1834±119^b^	41±4^a^	203±10^a^^b^	72±5^a^	24:69:7
**High**	Fiber-free	6	593±74^a^	146±26^a^	461±53^a^	225±37^a^	611±60^a^	495±99^a^	142±42^a^**	512±97^a^*	182±38^a^	36:39:25
	Pectin	13	904±129^a^*	298±51^a^*	1434±195^b^***	251±9^a^***	1959±108^b^***	111±132^a^	102±16^a^**	190±27^b^	110±15^b^	49:43:8
	Guar gum	21	1555±166^b^***	472±48^b^***	1452±108^b^***	301±22^a^***	2811±109^c^***	2202±129^b^	85±3^a^	255±20^b^	78±6^b^	38:58:4

^§^ Values are mean ± SEM.

^1^n, Number of animals.

^a,b,c^ Mean values within a column for different fat levels with different superscripts indicate a significant difference (P < 0.05).

*P < 0.05

**P < 0.01

***P < 0.001

mean values differed significantly from those of rats fed the corresponding low-fat diet.

^¥^ Primary BAs:group I secondary BAs:group II secondary BAs.

When molecular properties of guar gum and pectin were taken into consideration, the total amounts of primary BAs (CA, CDCA, and α-MCA) were higher in DF-containing diet groups than in the fiber-free control diet group, but significant differences (P < 0.05) were observed only for the three types of guar gum in the case of CA and for medium- and high-MW guar gum in the case of α-MCA (Table 3). Similarly, the total amounts of group I secondary BAs (UDCA, β-MCA, and ω-MCA) were generally higher in the cecum of rats fed DF as compared to those fed a fiber-free control diet. This was significant (P < 0.05) for guar gum-fed groups with respect to β-MCA and ω-MCA, and for LM pectin-fed groups with respect to ω-MCA. Groups fed guar gum had higher amounts of CA, α-MCA, and β-MCA than those fed HM pectin. Cecal levels of group II secondary BAs (LCA, DCA, and HDCA) were slightly lower in rats consuming DF-containing diets as compared to the fiber-free control diet, but only DCA was significantly lower (P < 0.05) in LM pectin-fed rats than in the control group (69 vs. 298 μg in the fiber-free control).

**Table 3:** BAs in the cecum of rats fed different types of pectin and guar gum in low- and high-fat diets (μg/cecum).§

Fat	Dietary fiber	Primary BAs			Group I secondary BAs			Group II secondary BAs		
		CA	CDCA	α-MCA	UDCA	β-MCA	ω-MCA	LCA	DCA	HDCA
**Low**	Fiber-free	174±30^b^	58±17^a^	189±20^c^	119±12^a^	465±133^c^	380±68^c^	54±11^a^	298±47^a^	101±14^a^
	LM pectin	321±40^a^^b^	123±3^a^	378±49^a^^b^^c^	161±7^a^	865±170^b^^c^	1446±545^a^^b^	46±11^a^	69±11^b^	66±19^a^
	HM pectin	229±85^b^	86±37^a^	180±59^c^	141±11^a^	479±58^c^	668±131^b^^c^	28±5^a^	201±62^a^^b^	72±20^a^
	Guar gum–low MW	545±112^a^	111±14^a^	308±71^b^^c^	147±9^a^	1437±129^a^	1319±193^a^^b^	45±10^a^	160±10^a^^b^	71±11^a^
	Guar gum–med MW	635±43^a^	125±20^a^	586±49^a^	128±15^a^	1317±110^a^^b^	2087±163^a^	30±4^a^	222±18^a^^b^	67±8^a^
	Guar gum–high MW	548±49^a^	148±9^a^	505±35^a^^b^	157±12^a^	1321±108^a^^b^	2096±110^a^	46±3^a^	226±11^a^	78±5^a^
**High**	Fiber-free	593±74^b^***	145±26^c^*	461±53^d^**	225±37^b^*	611±59^d^	495±99^c^	142±42^a^	511±97^a^	182±37^a^
	LM pectin	734±198^b^	231±89^c^	1886±318^a^^b^**	248±18^b^**	1842±95^c^***	1532±94^b^	91±19^a^	179±38^b^*	105±26^a^^b^
	HM pectin	1050±162^b^***	354±54^b^^c^**	1046±124^c^^d^***	235±10^b^***	2060±183^b^^c^***	750±106^c^	111±25^a^**	200±41^b^	114±18^a^^b^
	Guar gum–low MW	1779±271^a^**	487±76^a^^b^***	1261±47^b^^c^***	290±40^a^^b^**	2607±205^b^***	1466±26^b^	84±5^a^**	180±33^b^	61±11^b^
	Guar gum–med MW	2156±138^a^***	666±56^a^***	2041±90^a^***	384±28^a^***	3324±57^a^***	2666±46^a^**	84±5^a^***	246±22^b^	95±12^b^
	Guar gum–high MW	730±27^b^**	261±17^b^^c^***	1054±90^c^^d^***	228±17^b^**	2502±81^b^***	2474±157^a^	84±8^a^***	337±13^b^***	78±5^b^

^§^ Values represent mean ± SEM.

^a,b,c^ Mean values within a column for different fat levels with different superscripts indicate a significant difference (P < 0.05).

*P < 0.05

**P < 0.01

***P < 0.001

mean values differ significantly from those of rats fed the corresponding low-fat diet.

#### High-fat diets

When molecular properties of the diet were discounted, guar gum was associated with higher cecal levels of primary and group I secondary BAs (except for UDCA), and lower levels of group II secondary BAs (Table 2). Rats on a pectin diet had higher cecal levels of α-MCA and β-MCA and lower levels of DCA and HDCA. The distribution of BAs differed markedly between rats on the three diets. The contribution of group II secondary BAs was lower for the two DF groups (8% and 4% for pectin and guar gum, respectively) compared to the control group without DF (25%). On the other hand, the proportion of group I secondary BAs was higher for guar gum (58%) than for pectin (43%) and control (39%) diets.

The total amounts of primary BAs (CA, CDCA, and α-MCA) were higher in the low- and medium-MW guar gum-fed groups (1968 ± 205, 577 ± 66, and 1651 ± 69 μg, respectively) as compared to those fed the fiber-free control diet (593, 145, and 461 μg, respectively) (Table 3). The two pectin-fed groups had BA levels similar to those of the control group; only rats fed LM pectin had a higher amount of α-MCA (P < 0.05) than the DF-free control. The highest amounts of primary BAs were observed in rats fed medium-MW guar gum; these values were higher than in their high MW-fed counterparts (P < 0.05).

Of the group I secondary BAs (UDCA, β-MCA, and ω-MCA), the highest cecal levels of UDCA were in rats fed medium-MW guar gum (384 vs. 245 ± 24 μg for the other groups; P < 0.05); there were no other significant differences. The total amounts of β-MCA were higher in the cecum of rats fed all types of DF (2467 ± 124 μg) as compared to a fiber-free diet (611 μg; P < 0.05). The highest amount was in rats fed medium-MW guar gum (3324 μg), followed by high- and low-MW guar gum (2554 ± 143 μg; P < 0.05), and finally, the two pectins (1951 ± 139; P < 0.05). A similar trend was observed for ω-MCA, with the highest amounts measured in the medium- and high-MW guar gum groups (2570 ± 102 μg), followed by low-MW guar gum and LM pectin (1499 ± 60 μg, P < 0.05) and HM pectin and the fiber-free control diet (623 ± 103 μg, P < 0.05).

The total amounts of group II secondary BAs (LCA, DCA, and HDCA) were lower in the cecum of rats fed a DF as compared to a fiber-free control diet. DF had no effect on LCA but decreased the amount of DCA, whereas guar gum decreased the amount of HDCA in a MW-independent manner (P < 0.05).

#### Comparison between low- and high-fat diets

The total BA amounts were higher in the cecum of rats fed a high-fat as compared to a low-fat diet, although the differences were not always significant (Tables 2 and 3). The largest differences in DF groups were observed for CA, CDCA, α-MCA, UDCA, β-MCA, and LCA levels. The proportion of primary BAs was lower with a low-fat than with a high-fat diet (24% vs. 36%–49%), while the proportion of group I secondary BAs was higher.

#### Correlation between BAs in the cecum and cholesterol and triglyceride levels in blood and liver

Data for cholesterol and triglyceride levels in portal blood as well as in the liver were obtained from another study of metabolic responses [24]; the biomarkers are summarized in Fig 1. Strong positive correlations were detected between cecal levels of LCA, DCA, and HDCA and cholesterol levels in portal plasma (r = 0.57, 0.33, and 0.35, respectively) and liver (r = 0.62, 0.32, and 0.38, respectively), which was independent of diet (S1 Fig).

Cecal levels of LCA and DCA was also positively correlated with triglyceride levels in plasma and liver (S2 Fig) and the r-values in portal plasma were 0.35 and 0.49 (p<0.01 and p< 0.001, respectively) and corresponding values in the liver were 0.44 and 0.36, respectively (both p< 0.001). There was no correlation between HDCA and triglycerides in the liver and plasma.

### Inflammatory marker levels in blood

Expression of inflammatory marker LBP was generally elevated in rats on a high-fat as compared to a low-fat diet (53 ± 9 vs. 33 ± 5 ng/ml). More specifically, high-fat groups had higher LBP levels (P < 0.05) than corresponding low-fat DF groups fed LM pectin (57 ± 17 vs. 35 ± 11 ng/ml), low-MW guar gum (57 ± 16 vs. 35 ± 16 ng/ml), and high-MW guar gum (52 ± 4 vs. 41 ± 9 ng/ml). However, there were no significant differences in LBP levels between low- and high-fat groups fed medium-MW guar gum and HM pectin, indicating that these DFs countered the high-fat-induced increase in LBP levels.

### Effect of medium-MW guar gum and HM pectin on gut microbiota composition in rats fed a high-fat diet

**Fig 2:**
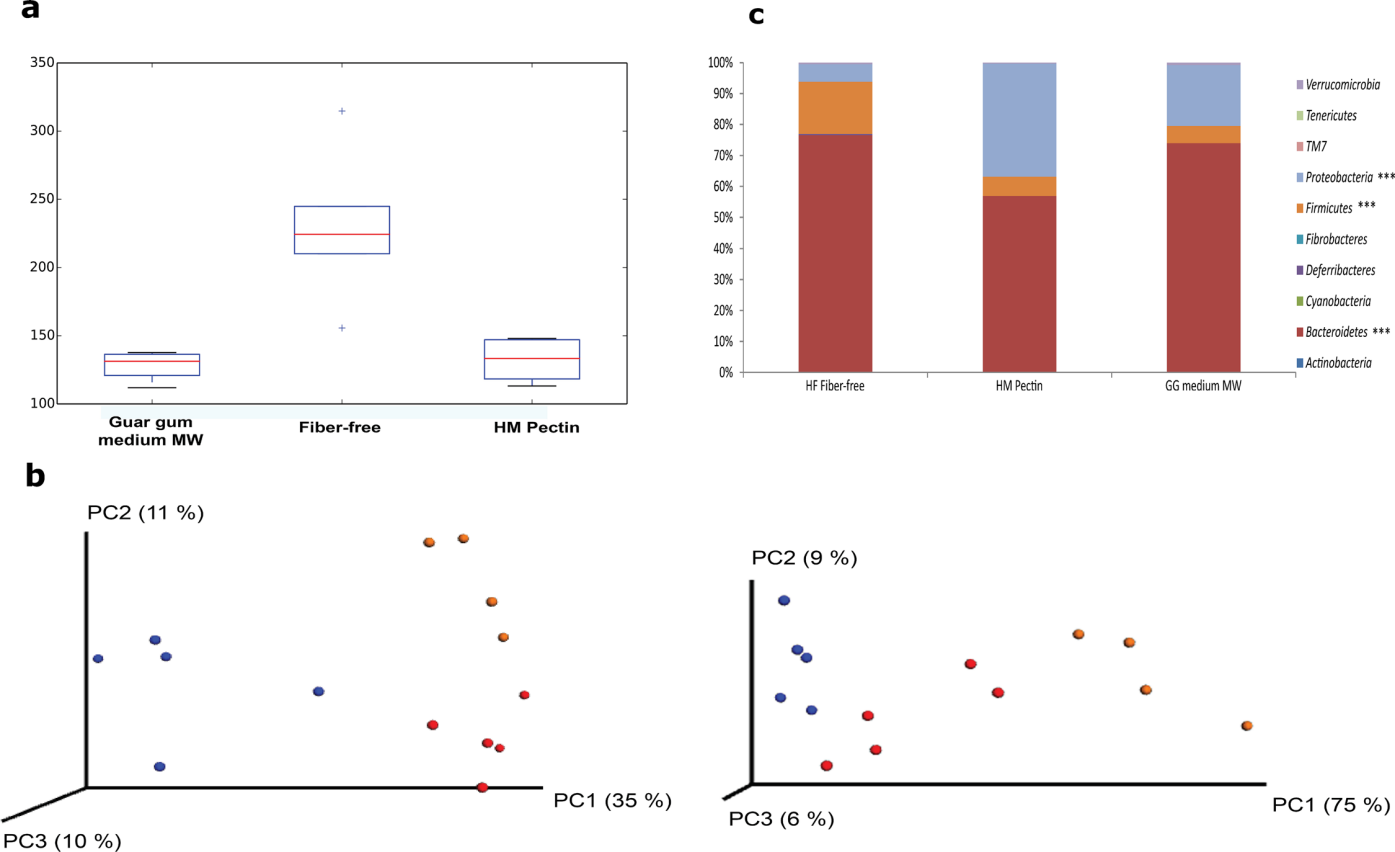
Alpha diversity and mean relative abundance of bacterial taxa at the phylum level in rats fed HM pectin (n = 4), medium-MW guar gum (n = 5), or a fiber-free diet (n = 5) in a high-fat setting. (a) Diversity was greater in the fiber-free control than in the fiber-fed groups (HM-pectin vs. fiber-free, P < 0.05; guar gum of medium-MW vs. control, P < 0.01). (b) Principle component analysis of 16S rRNA gene profiles for unweighted (left) and weighted (right) UniFrac metrics to estimate phylogenetic distance between groups (based on sub-sampled datasets of 66,905 sequences/sample). Blue = control, red = guar gum of medium–MW, and orange = HM-pectin group. (c) Bacteroidetes abundance was lower in the HM-pectin than in the other two groups (P < 0.001). Firmicutes abundance was higher in the fiber-free as compared to the DF groups (P < 0.001), while Proteobacteria abundance differed significantly among all three groups (P < 0.001).

**Fig 3:**
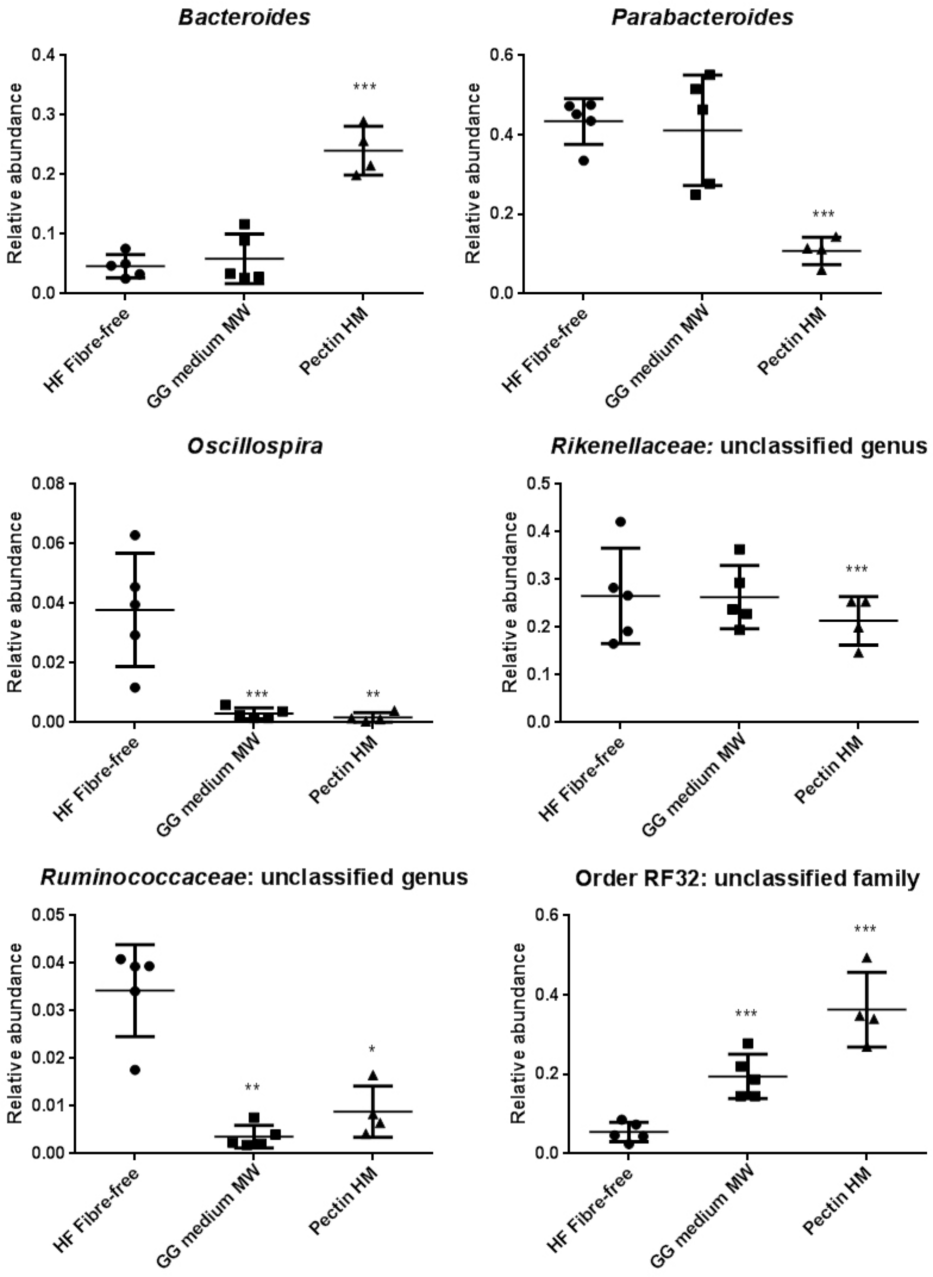
Mean relative abundance of bacterial taxa at the genus level in rats fed HM pectin (n = 4), medium-MW guar gum (n = 5), or a fiber-free diet (n = 5) in a high-fat setting. Data represent mean ± standard deviation for bacterial genera. *P < 0.05, **P < 0.01, ***P < 0.001, fiber groups vs. control (one-way ANOVA and Tukey’s test for multiple comparisons).

Sequencing of bacterial 16S rRNA genes from the cecum of rats in the guar gum medium-MW, HM pectin, and fiber-free control groups in the high-fat setting revealed that alpha-diversity was significantly lower in both fiber groups than in the control group (medium MW guar gum, P < 0.01 and HM pectin, P < 0.05; observed species test, QIIME) (Fig 2A). Both unweighted and weighted UniFrac distance metrics showed clustering of the groups in a principal coordinate analysis (Fig 2B). The diets altered the representation of Bacteroidetes, Firmicutes, and Proteobacteria phyla (Fig 2C); notably, the relative abundance of Bacteroidetes was lower in the HM pectin group than in the other two groups (P < 0.001). Firmicutes was more highly represented in the fiber-free control as compared to the DF groups (P < 0.001), while Proteobacteria abundance differed across all three groups (P < 0.001). At the genus level, the abundances of *Bacteroides*, *Parabacteroides*, and *Oscillospira*; unclassified genera in the *Rikenellaceae* and *Ruminococcaceae* families; and an unclassified family in *RF32* differed between groups (Fig 3). Additional details on the relative abundance of bacterial taxa at the phylum and genus levels in the different groups are shown in Table B in S1 File.

### SCFAs

The amounts of acetic, propionic, butyric, and caproic acid were higher in groups fed guar gum and HM pectin than in groups fed a fiber-free control diet. Rats fed guar gum also had higher amounts of propionic acid than those that consumed the other diets (Table 4).

**Table 4:** SCFAs in the cecum of rats fed medium-MW guar gum, HM pectin, or a fiber-free control diet in a high-fat setting (μmol/cecum).§

Diet/SCFA	n^1^	Acetic	Propionic	Iso-butyric	Butyric	Iso-valeric	Valeric	Caproic
**Control**	5	120±14^a^	21±4^a^	3±0.7^a^	15±3^a^	4±0.9^a^	2±0.9^a^	0.1±0.02^a^
**HM pectin**	4	446±33^b^	49±10^b^	4±0.3^a^	25±4^a^^,^^b^	4±0.5^a^	1±0.1^a^	0.5±0.02^b^
**Medium-MW guar gum**	5	539±16^c^	112±6^c^	4±0.4^a^	28±2^b^	5±0.4^a^	1±0.1^a^	0.5±0.04^b^

^§^ Values represent mean ± SEM.

^1^n, Number of animals.

^a,b,c^ Mean values within a column with different subscript letters differed significantly (P < 0.05).

### Multivariate analysis of BAs, LBP, SCFAs, and microbiota

**Fig 4:**
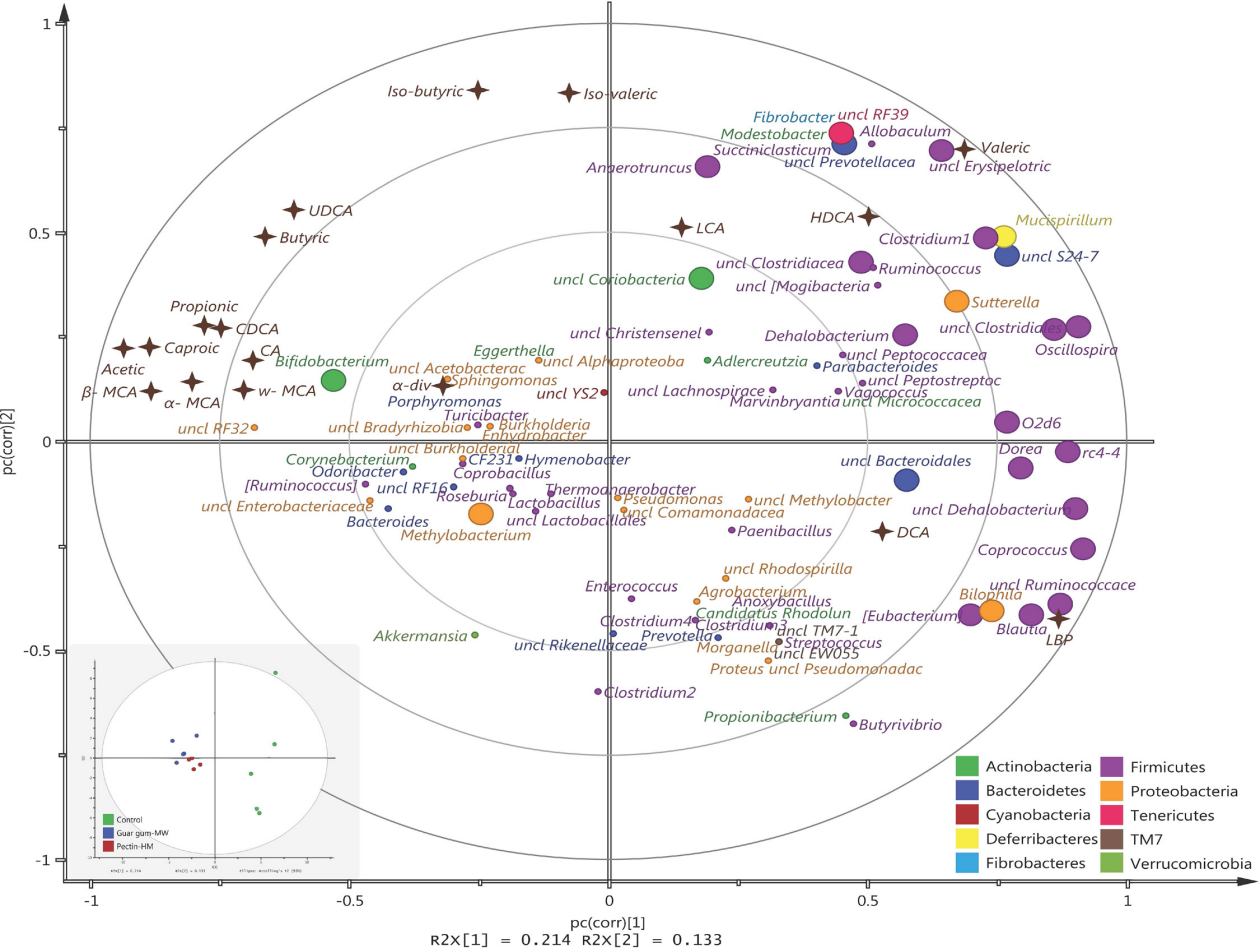
PLS loading and score scatter plots of different biomarkers. Plots illustrate correlations between the biomarkers (brown stars) and the gut microbiota (circles) in rats fed medium-MW guar gum, HM pectin, or a fiber-free control diet in a high-fat setting. Gut microbial taxa are colored according to the phylum to which they belong (dark green = Actinobacteria; dark blue = Bacteroidetes; red = Cyanobacteria; yellow = Deferribacteres; light blue = Fibrobacteres; purple = Firmicutes; orange = Proteobacteria; pink = Tenericutes; brown = TM7; light green = Verrucomicrobia). Variables situated close or opposite to each other are positively and negatively correlated, respectively. A larger distance from the origin (0,0) indicates a stronger correlation. Bacterial genera were correlated with the biomarkers shown in large circles. The score scatter plot to the bottom left shows how each rat is situated with respect to the PLS loading plot and according to groups (green = fiber-free control; blue = medium-MW guar gum; red = HM pectin). The Clostridium genera (1–4) were from different families: *Clostridium1* was from *Clostridiaceae*, *Clostridium2* was from *Lachnospiraceae*, *Clostridium3* was from *Peptostreptococcaceae*, and *Clostridium4* was from *Ruminococcaceae*.

To assess the relationships between BA profiles, LBP, SCFAs, and gut microbiota, we carried out a PLS analysis in rats that consumed HM pectin, medium-MW guar gum, and a fiber-free control diet with high-fat content to determine how different types of BAs are related to microbiota, inflammatory marker expression, and SCFA levels. PLS loading and score scatter plots (Fig 4) indicated that CA, CDCA, UDCA, α-MCA, β-MCA, and ω-MCA (i.e., primary BAs and group I secondary BAs) formed one cluster and HDCA and LCA (group II secondary BAs) formed another. DCA was separate from both clusters. Most SCFAs—with the exception of valeric acid—clustered together with primary and group II secondary BAs and were higher in the DF groups as compared to the control. HDCA and LCA were associated with valeric acid whereas LBP was associated with DCA; the levels of all of these BAs were higher in rats fed a fiber-free control diet. Primary and secondary BAs from group I and all SCFAs except for valeric acid were positively correlated with *Bifidobacterium* and also *RF32*. On the other hand, group II secondary BAs (DCA, LCA, and HDCA) were negatively correlated with those biomarkers, but positively correlated with most members from Firmicutes (e.g., *Oscillospira*, rc4-4, *Coprococcus*, unclassified genera in families *Dehalobacteriaceae*, *Ruminococcaceae*, *Eubacterium*, and an unclassified family in *Clostridiales*) (Table C in S1 File). Furthermore, *Dorea*, *Blautia*, *Ruminococcaceae*, and *Bilophila* were positively correlated with plasma LBP and negatively correlated with all SCFAs except for valeric acid. LCA, HDCA, and valeric acid were positively correlated with *Fibrobacter*, *Modestobacter*, *Succiniclasticum*, *Allobaculum*, *Anaerotruncus*, *Oscillospira*, and *Dehalobacterium*; *Clostridium* from the family *Clostridiaceae*; unclassified genera in the *Prevotellaceae*, *Erysipelotrichaceae*, *S24-7*, and *RF39* families; and an unclassified family in *Clostridiales*. LCA was positively correlated with an unclassified genus in *Coriobacteriaceae*.

The alpha diversity reflects species richness within a sample and was found to be positively correlated with *Bifidobacterium* and *Methylobacterium*, and clustered with primary and group I secondary BAs.

## Discussion

In the present study, cecal BA profiles were analyzed in rats fed guar gum or pectin—which are highly soluble and fermented by gut microbiota—in the context of a low- or high-fat diet. Guar gum with different chain lengths and pectins with different degrees of methoxylation were used to determine whether the molecular properties of DF influence BA profiles. The MW of DFs—and thus, their viscosity—plays an important role in their metabolic effects, which are greater for more viscous fibers. It has been suggested that viscous DFs trap BAs in the upper intestinal tract, thereby diminishing their absorption and lowering cholesterol levels [37]. On the other hand, a lower MW and degree of methoxylation decreases viscosity, which may diminish the hypocholesterolemic effect of fibers. Pectin may also bind BAs to different degrees depending on environmental pH and degree of methoxylation. None of these parameters have been previously investigated in relation to BAs.

Among rats on a high-fat diet, medium-MW guar gum was the most effective in increasing the amount of primary BAs (CA, CDCA, and α- MCA) and group I secondary BAs (UDCA, β-MCA, and ω-MCA) in cecum. These changes in BAs corresponded with the level of the inflammatory marker LBP, which did not increase in rats fed medium-MW guar gum along with a high-fat diet relative to the corresponding low-fat group. However, the changes were not observed with any other type of the DFs investigated. Concerning Group II secondary BAs they instead decreased in rats fed medium-MW guar gum compared with the fiber-free control; low- and high-MW guar gum also decreased the amount of HDCA and DCA, while the two types of pectin only decreased the amount of DCA in cecum. In this respect, it is worth noting that medium-MW guar gum yielded a BA profile with higher amounts of primary and group I secondary BAs than the other types of guar gum, possibly because it generates higher levels of butyric acid during fermentation, which has been linked to the prevention of hyperlipidemia and liver steatosis (Fig 1) [24]. The importance of MW for the SCFA profile has also been shown by others [25, 26]. In fact, low-MW fructo-oligosaccharides and arabinoxylans are more likely to form butyric acid than their high-MW counterparts [25, 26]. Interestingly, microbiota composition also varies according to the MW of arabinoxylans [26]. Although the underlying mechanism is not well understood, an explanation may be an interaction between the fermentation and the viscosity during the formation of SCFAs, which in turn influences also the microbiota composition. The influence of MW on microbiota composition and gut metabolite formation has been further demonstrated with barley degraded to lower-MW derivatives by malting [38]. However, the possibility that DFs with high viscosity and high MW may incorporate digestible components into their network and deliver them to the colon as substrates for microbiota cannot be discounted.

The MW of guar gum did not greatly affect BA composition in the low-fat setting, with rats fed different types of guar gum exhibiting similar but higher amounts of some BAs (CA, α-MCA, β-MCA, and ω-MCA) in the cecum than their counterparts that consumed a fiber-free control diet. This is interesting since it is supposed that higher viscosity has a greater effect on cholesterol levels [39]. However, if fermentation is a mechanism of importance this is also expected since high-MW guar gum is completely degraded by microbiota, thereby abolishing its viscosity and binding capacity. Notably, the level of BAs—which are presumed to improve host health—was higher in groups fed guar gum as compared to pectin; this may be explained by the fact that guar gum generates propionic acid, an SCFA that has positive effects on cholesterol [8]. The total amounts of CDCA and UDCA—which may have health-promoting effects in the host—were unaffected by any of the DFs in the low-fat setting. UDCA has been reported to reduce mucosal permeability and consequently, block the transfer of toxic and inflammatory compounds through the mucosa [15].

The total amounts of LCA, DCA, and HDCA in the cecum of rats were correlated with cholesterol and triglyceride levels in the liver and portal plasma. These results show that BA levels can be reduced by consuming a low-fat diet. We also observed correlations between primary and group II secondary BAs and specific SCFAs and microbial taxa. Interestingly, group II BAs and LBP were mainly associated with bacteria from phylum Firmicutes, while all SCFAs as well as primary and group I secondary BAs were associated with Bifidobacterium, which includes several species that are probiotics [40] (Fig 4). However, these associations between specific microbes and other biomarkers may not be direct, since the gut microbial ecosystem is complex and bacteria may act co-dependently via production of different metabolites.

HDCA level was positively correlated with valeric acid content and various bacterial taxa. It was previously reported that patients with microscopic colitis had higher fasting serum valeric acid levels than healthy controls, with no other differences in serum SCFAs [41]. This is interesting since the levels of SCFAs formed during fermentation in the lower gut tend to be correlated with those in blood [42]. In this context, it is important to note that a positive correlation was detected between ulcer area and fecal HDCA concentration in rats with dextran sulfate sodium-colitis [43], while an in vitro study showed that HDCA was cytotoxic despite its hydrophilic properties [18]. Future studies should clarify the effects of different types of fiber-containing foods—rather than individual DFs—on BA and SCFA profiles. For instance, whole-grain barley processed to barley malt altered gut microbiota composition and SCFA profile in rats [38]; the BA composition is currently under investigation. Pectin and guar gum are mostly used in small amounts as thickeners in foods and as a replacement for fat in low-calorie products. An analysis of BA profiles in blood rather than in feces only could provide additional insight into the effects of different types of foods on cholesterol elimination and lipid absorption.

In conclusion, high-fat diets containing DF had a greater effect than low-fat diets on the total amount of BAs, which was also dependent on the molecular properties of DFs. Medium- and to some extent, low-MW guar gum increased the amount of primary and group I secondary BAs and reduced the amount of DCA and HDCA in the cecum. In addition, medium-MW guar gum abolished the high-fat diet-induced increase in the expression of the inflammatory marker LBP. The effects of pectin with varying degrees of methoxylation were less pronounced than those of guar gum. However, if comparing the two pectins, effects with HM were more distinct than with LM. Finally, the molecular properties of the fibers were associated with the abundance of specific microbial genera and gut BA profiles. These findings may suggest that modifying the molecular properties of fibers can be an effective dietary intervention for the prevention or treatment of metabolic disorders.

## Supporting Information

S1 Fig Correlation between cholesterol and secondary BAs. (a–c) Correlation between plasma cholesterol and cecal levels of LCA (r = 0.57; P < 0.001) (a), DCA (r = 0.33; P < 0.01) (b), and HDCA (r = 0.35; P < 0.01) (c). (d–f) Correlation between liver cholesterol and cecal levels of LCA (r = 0.62; P < 0.001) (d), DCA (r = 0.32; P < 0.01) (e), and HDCA (r = 0.38; P < 0.001) (f).
(TIF)

S2 Fig Correlation between triglyceride and secondary BA levels. (a, b) Correlation between plasma triglyceride levels and cecal levels of LCA (r = 0.35; P < 0.01) (a) and DCA (r = 0.49; P < 0.001) (b). (c, d) Correlation between liver triglyceride levels and cecal levels of LCA (r = 0.44; P < 0.001) (c) and DCA (r = 0.36; P < 0.001) (d). There was no correlation between triglyceride and HDCA levels in the portal plasma and liver.
(TIF)

S1 File Supporting tables. This file contains Table A, showing composition of experimental diets (g/kg dwb). Table B, showing Mean relative abundance of bacterial taxa at phylum and genus level in rats fed high-methoxylated pectin (n = 4), medium molecular weight guar gum (n = 5), or fiber-free diet (n = 5) in a high-fat setting. Table C, showing P-values for significant correlations between different biomarkers and the gut microbiota (adjusted p-values using Benjamini-Hochberg procedure).
(DOCX)
