# Supplementary material for: Molecular Properties of Guar Gum and Pectin Modify Cecal Bile Acids, Microbiota, and Plasma Lipopolysaccharide-Binding Protein in Rats
Source: PLoS One. 2016 Jun 17;11(6):e0157427. doi: 10.1371/journal.pone.0157427 (PMC4912110; doi:10.1371/journal.pone.0157427)
Supplement: S1 File — This file contains Table A, showing composition of experimental diets (g/kg dwb). Table B, showing Mean relative abundance of bacterial taxa at phylum and genus level in rats fed high-methoxylated pectin (n = 4), medium molecular weight guar gum (n = 5), or fiber-free diet (n = 5) in a high-fat setting. Table C, showing P-values for significant correlations between different biomarkers and the gut microbiota (adjusted p-values using Benjamini-Hochberg procedure). (DOCX) [file pone.0157427.s003.docx]

Supporting Information

Table A. Composition of experimental diets (g/kg dwb)

|  | Low-fat | | | High-fat | | |
| --- | --- | --- | --- | --- | --- | --- |
|  | Pectin | Guar gum | Control | Pectin | Guar gum | Control |
| Basic formulation^1^  *of which rapeseed oil* | 409  *50* | 409  *50* | 409  *50* | 409  *50* | 409  *50* | 409  *50* |
| Lard | - | - | - | 230 | 230 | 230 |
| Cholesterol | - | - | - | 20 | 20 | 20 |
| Wheat starch^¥^ | 491 | 503 | 591 | 241 | 253 | 341 |
| Pectin^ψ^ or Guar gum^§^ | 100 | 88 | - | 100 | 88 | - |

1 Containing (g/kg, dwb): 200 protein (casein from Sigma Aldrich, St. Louis, MO, USA), 50 rapeseed oil (Zeta, Stockholm, Sweden), 1.2 DL-methionine (Sigma Aldrich, St. Louis, MO, USA), 100 sucrose (Nordic sugar, Copenhagen, Denmark), 8 vitamin mixture†, 2 choline chloride (Sigma Aldrich, St. Louis, MO, USA), 48 mineral mixture‡

†Containing (g/kg): 0.62 menadione, 2.5 thiamin hydrochloride, 2.5 riboflavin, 1.25 pyridoxine hydrochloride, 6.25 calcium pantothenate, 6.25 nicotinic acid, 0.25 folic acid, 12.5 inositol, 1.25 p-aminobenzoic acid, 0.05 biotin, 0.00375 cyanocobalamin, 0.187 retinol palmitate, 0.00613 calciferol, 25 d-α-tocopheryl acetate, 941.25 maize starch (Lantmännen, Stockholm, Sweden)

‡ Containing (g/kg): 0.37 CuSO_4_·5H_2_O, 1.4 ZnSO_4_·7H_2_O, 332.1 KH_2_PO_4_, 171.8 NaH_2_PO_4_·2H_2_O, 324.4 CaCO_3_, 0.068 KI, 57.2 MgSO_4_, 7.7 FeSO_4_·7H_2_O, 3.4 MnSO_4_·H_2_O,

0.02 CoCl·6H_2_O, 101.7 NaCl, 0.019 chromium(III)chloride and 0.011 sodiumselenate.

¥ Norfoods Sweden AB, Malmö, Sweden, varied according to the dietary fiber content of the test materials.

Ψ Containing 80 g/kg fiber (dwb)

§ Containing 91 g/kg fiber (dwb)

Table B. Mean relative abundance of bacterial taxa at phylum and genus level in rats fed high-methoxylated pectin (n = 4), medium molecular weight guar gum (n = 5), or fiber-free diet (n = 5) in a high-fat setting. Bacteroidetes abundance was lower in the HM-pectin group as compared with the other two groups (***P < 0.001). Firmicutes was higher in the fiber-free group compared with the fiber groups (P < 0.001), while Proteobacteria differed significantly between all three groups (P < 0.001). At genus level, six genera differed significantly between the fiber groups and the fiber-free control group, as indicated by *P < 0.05, **P < 0.01, and ***P < 0.001. Differences were calculated by one-way ANOVA with Tukey’s test for multiple comparisons.

| Phylum | HF Fiber-free | HM Pectin | GG medium MW |
| --- | --- | --- | --- |
| Actinobacteria | 1.66E-04 | 5.76E-05 | 1.79E-04 |
| Bacteroidetes | 0.77 | 0.60 *** | 0.74 |
| Cyanobacteria | 1.82E-06 | 0 | 3.60E-06 |
| Deferribacteres | 4.24E-03 | 3.59E-05 | 3.59E-05 |
| Fibrobacteres | 1.68E-06 | 0 | 0 |
| Firmicutes | 0.17 | 0.063*** | 0.056*** |
| Proteobacteria | 0.057 | 0.37*** | 0.20*** |
| TM7 | 6.80E-06 | 0 | 0 |
| Tenericutes | 5.03E-06 | 0 | 0 |
| Verrucomicrobia | 5.14E-03 | 3.68E-03 | 7.67E-03 |
|  |  |  |  |
| Genus | HF Fiber-free | HM Pectin | GG medium MW |
| *Parabacteroides* | 0.434 | 0.108*** | 0.411 |
| *Rikenellaceae_uncl genus* | 0.266 | 0.214*** | 0.263 |
| *Clostridiaceae_uncl genus* | 0.034 | 0.012 | 0.022 |
| *Bacteroides* | 0.046 | 0.240*** | 0.059 |
| *Ruminococcaceae_uncl genus* | 0.034 | 0.009* | 0.004** |
| *S24-7_uncl genus* | 0.019 | 0.007 | 0.006 |
| *Oscillospira* | 0.038 | 0.002** | 0.003*** |
| *Lachnospiraceae_uncl genus* | 0.012 | 0.016 | 0.005 |
| *RF32_uncl family* | 0.055 | 0.363*** | 0.195*** |
| *Clostridium* | 0.007 | 0.001 | 0.000 |
| *Blautia* | 0.010 | 0.001 | 0.001 |
| Other < 1 % | 0.045 | 0.027 | 0.031 |

Table C. P-values for significant correlations between different biomarkers and the gut microbiota (adjusted p-values using Benjamini-Hochberg procedure).

|  | |  | |  | | |  | |  | |  | |  | |  | | |  | |  | |  | |  |
| --- | --- | --- | --- | --- | --- | --- | --- | --- | --- | --- | --- | --- | --- | --- | --- | --- | --- | --- | --- | --- | --- | --- | --- | --- |
| Bacterial genera | LCA | | β-MCA | | | HDCA | | | LBP | | | α-div | | Acetic acid | | Propionic acid | | | Butyric acid | | Valeric acid | | Caproic acid | |
| *Eubacterium* | - | | - | | | - | | | - | | | - | | <0.05(neg) | | - | | | - | | - | | <0.05(neg) | |
| *02d06* | - | | - | | | - | | | - | | | - | | <0.05(neg) | | - | | | - | | - | | - | |
| *Allobaculum* | - | | - | | | <0.05(pos) | | | - | | | - | | - | | - | | | - | | <0.001(pos) | | - | |
| *Anaerotruncus* | - | | - | | | <0.05(pos) | | | - | | | - | | - | | - | | | - | | - | | - | |
| uncl family *Bacteroidales* | - | | - | | | - | | | <0.05(pos) | | | - | | - | | - | | | - | | - | | - | |
| *Bifidobacterium* | - | | - | | | - | | | - | | | <0.001(pos) | | - | | - | | | - | | - | | - | |
| *Bilophila* | - | | - | | | - | | | - | | | - | | <0.01(neg) | | - | | | - | | - | | <0.05(neg) | |
| *Blautia* | - | | - | | | - | | | <0.05(pos) | | | - | | <0.001(neg) | | - | | | <0.05(neg) | | - | | <0.001(neg) | |
| uncl genus *Clostridiaceae* | - | | - | | | - | | | - | | | - | | - | | - | | | - | | <0.05(pos) | | - | |
| *Clostridiales* | - | | <0.05(neg) | | | - | | | <0.001(pos) | | | - | | <0.01(neg) | | - | | | - | | <0.01(pos) | | <0.001(neg) | |
| *Clostridium* | - | | | | - | | | - | | - | | - | | - | | | - | | - | | <0.001(pos) | | - | |
| *Coprococcus* | - | | | | <0.05(neg) | | | - | | <0.05(pos) | | - | | <0.001(neg) | | | - | | - | | - | | <0.001(neg) | |
| uncl genus *Coriobacteriaceae* | <0.001(pos) | | | | - | | | - | | - | | - | | - | | | - | | - | | - | | - | |
| uncl genus *Dehalobacteriaceae* | - | | | | <0.001(neg) | | | - | | - | | - | | <0.001(neg) | | | - | | - | | - | | <0.001(neg) | |
| *Dehalobacterium* | - | | | | - | | | - | | - | | - | | - | | | - | | - | | <0.05(pos) | | - | |
| *Dorea* | - | | | | - | | | - | | <0.001(pos) | | - | | <0.01(neg) | | | - | | - | | - | | <0.05(neg) | |
| uncl genus *Erysipelotrichaceae* | - | | | | - | | | <0.05(pos) | | - | | - | | - | | | - | | - | | <0.001(pos) | | - | |
| *Fibrobacter* | - | | | | - | | | <0.001(pos) | | - | | - | | - | | | - | | - | | <0.01(pos) | | - | |
| *Methylobacterium* | - | | | | - | | | - | | - | | <0.001(pos) | | - | | | - | | - | | - | | - | |
| *Modestobacter* | - | | | | - | | | <0.001(pos) | | - | | - | | - | | | - | | - | | <0.01(pos) | | - | |
| *Mucispirillum* | - | | | | - | | | - | | <0.05(pos) | | - | | - | | | - | | - | | <0.001(pos) | | - | |
| *Oscillospira* | - | | | | <0.05(neg) | | | - | | <0.05(pos) | | - | | <0.01(neg) | | | - | | - | | <0.05(pos) | | <0.05(neg) | |
| uncl genus *Prevotellaceae* | - | | | | - | | | <0.05(pos) | | - | | - | | - | | | - | | - | | <0.05(pos) | | - | |
| *rc4-4* | - | | | | <0.05(neg) | | | - | | <0.05(pos) | | - | | <0.001(neg) | | | - | | - | | - | | <0.001(neg) | |
| uncl genus *RF32* | - | | | | - | | | - | | - | | - | | - | | | - | | - | | - | | <0.05(pos) | |
| *RF39* | - | | | | - | | | <0.001(pos) | | - | | - | | - | | | - | | - | | <0.01(pos) | | - | |
| uncl genus *Ruminococcaceae* | - | | | | <0.001(neg) | | | - | | <0.05(pos) | | - | | <0.001(neg) | | | <0.001(neg) | | <0.001(neg) | | <0.001(pos) | | <0.001(neg) | |
| uncl genus *S24-7* | - | | | | - | | | - | | <0.05(pos) | | - | | - | | | - | | - | | - | | - | |
| *Succiniclasticum* | - | | | | - | | | <0.001(pos) | | - | | - | | - | | | - | | - | | <0.01(pos) | | - | |
| *Sutterella* | - | | | | - | | | <0.05(pos) | | - | | - | | - | | | - | | - | | - | | - | |

−, No correlation.

Uncl, Unclassified.
